# Aerotoxic Syndrome—Susceptibility and Recovery

**DOI:** 10.3390/toxics13060420

**Published:** 2025-05-22

**Authors:** Jeremy J. Ramsden

**Affiliations:** 1 Department of Biomedical Research, Faculty of Medicine and Health Sciences, The University of Buckingham, Buckingham MK18 1EG, UK; j.ramsden@colbas.org; 2 Sustainable Aviation Unit, Collegium Basilea (Institute of Advanced Study), 4053 Basel, Switzerland

**Keywords:** aircraft maintenance, carbon monoxide, deregulation, fume events, inhalation toxicity, tricresyl phosphate

## Abstract

Significant numbers of aircrew and jet airline passengers are affected by post-flight symptoms of ill health, usually nowadays labelled “aerotoxic syndrome”. It could be inferred from a large passenger survey carried out in the Netherlands that up to 50% of flights may engender malaise to varying degrees, and up to 50% of the population might be susceptible to suffering from actual intoxication from the contaminants known to occur in aircraft cabin air. In-flight measurements of its composition have revealed the presence of known neurotoxins, notably tricresyl phosphate and carbon monoxide, both of which can enter the cabin air as it is bled off the main engines. This study reviews the quantitative aspects of this evidence and estimates the susceptibility of the population to neurological damage at the measured levels of contamination, its typical impacts on health, and the likelihood and timescales of post-exposure recovery. Airworthiness directives already mandate that crew and passenger compartment air must be free from harmful or hazardous vapours and gases, but uncertainty regarding the nature of these particular hazards has led to this important aspect of airworthiness having been hitherto unduly neglected. The continuing exponential growth of air passenger traffic means that cabin air contamination will eventually become a major public health hazard if effective action is not taken, some possible courses of which are discussed.

## 1. Introduction

Insofar as flying in heavier-than-air machines is an unnatural activity, flyers must be prepared to consider possible adverse health impacts, much as in other unnatural activities such as travel to outer space, deep sea diving and submarining. Most of these other activities have stringent fitness requirements imposed on participants, whereas in flying, only aircrew must pass regular medical examinations in order to be permitted to continue with the activity. In the early days of railways, commentators warned of the possibility of adverse health effects from travel at high speed and through tunnels, but it quickly became apparent that the effects were negligible for the vast majority of participants. Particular features of modern jet airliners that may adversely impact health include enhanced exposure to cosmic radiation at typical cruise altitudes of around 10 km above sea level, elevated ozone concentrations associated with the “ozone layer” [1,2], confinement for prolonged periods with potential carriers of infectious pathogens, and breathing air bled off the main engines and therefore possibly contaminated with substances associated with their operation.

The effects of cosmic rays have been dealt with elsewhere and will not be further discussed here. Similarly, the enhanced risk of microbial infection has also been discussed elsewhere [3,4]; aircraft cabin air flow (usually comprising HEPA filtration of recirculated air) is designed to minimize the presence of microbes in the atmosphere. The risks associated with breathing bleed air have been far less investigated. Milestones were the BALPA (British Airline Pilots Association) Air Safety and Cabin Air Quality International Aero Industry conference in 2005 in London [5] and the Inhalable Toxic Contamination in Aircraft Cabin Air (ITCOBA) workshop in 2011 at Cranfield University, UK [6]. The problem is relatively new because (a) early large passenger aircraft did not fly high enough to require pressurization and (b) when pressurization came to be needed, it was supplied by compressing air taken directly from the exterior [7]. Some military aircraft flying at high altitudes were pressurized quite early on by bleeding compressed air from the jet engines, and the potential health risks through contamination, especially with engine oil, were recognized, but pilots were provided with a personal air supply (e.g., from small tanks) to eliminate the risk. Bleed air in passenger airliners was introduced in the 1960s and soon almost all aircraft were equipped with it. A significant event was the 1978 USC deregulation act [8,9], which suddenly imposed severe cost constraints on airline operations, encouraging the deferral of maintenance perceived to be nonessential, including of the bleed air system.

The term “aerotoxic syndrome” (AS) was introduced by Balouet and Winder more than two decades ago to describe the set of symptoms apparently increasingly being suffered by aircrew [10]. Given the necessary presence of the known neurotoxin [11] tricresyl phosphate (TCP) in jet engine lubricating oil [12] (it is an essential metal-passivating antiwear agent) and the characteristics of the oil seals functioning to keep oil out of the bleed air, the design features of which make a small amount of leakage inevitable [13,14,15], TCP has long attracted scrutiny as a possible causative agent of AS [16,17,18]. Less scrutiny has hitherto been given to carbon monoxide, although its dangers in general aviation have long been apparent, with warnings and guidance on how to minimize them having been issued [19]. Perhaps surprisingly, CO has only recently been considered to be a problem in jetliner cabins and a potential contributor to AS [20], even though the mechanisms for its generation in the engines and pathways for its transfer into cabin air are known to exist [21] (cigarette smoking would also have generated CO in the cabin but smoking is now generally banned on flights). Even less scrutiny has been accorded to fumes emanating from leaking hydraulic fluid, possibly because its principal constituent is tributyl phosphate (TBP), which is not considered to be neurotoxic; nevertheless, He et al. concluded that hydraulic fumes were more toxic than engine oil fumes because TBP has been found to have a much higher concentration in the cabin than does TCP [22].

A very comprehensive survey of frequent flyers (n=500, drawn from an initial group of over 8000; 62% were male) in the Netherlands found that slightly more than 50% had no health-related complaints during or after flying. Concomitantly, over 40% reported some malaise following a jetliner flight [23], a remarkably high prevalence. The two extreme interpretations of this finding are *either* that the entire population is susceptible to malaise-engendering cabin air contamination, which occurs on about half of flights, *or* that about half of the population is susceptible to malaise-engendering cabin air contamination, which is present in all flights. In Section 3 and Section 5 we shall explore to what extent independent empirical findings of contamination and susceptibility are compatible with these data.

## 2. The Nature of Aerotoxic Syndrome

One of the problems with diagnosing AS is that its most characteristic symptoms [24,25,26]—such as chest pains, eye irritation and headache—are widely found in the general population. Nevertheless, even though it is highly plausible to ascribe their occurrence individually to random causes, the simultaneous occurrence of, say, five or more of these characteristic symptoms cannot be plausibly ascribed to chance [18], at least not in a completely randomly chosen population. On the other hand, there are inevitably some correlations between the passengers on any particular flight—they have all started their journey in the same place, where there might happen to be widespread respiratory infection, and all suffer from mild hypoxia on an aircraft (cabins are not pressurized to sea level), both of which might lead to multiple symptoms commonly associated with AS but not engendered by the chemical contamination of cabin air. As is apparent from the large passenger survey [23], few sufferers seek medical advice for their malaise; a common workaround is to allow an extra day in a travel schedule to permit recovery. Hence, the symptoms of many presumed sufferers are unreported, which makes it difficult to reach an unambiguous analysis of possible causes.

Far more serious is the situation with aircrew, with whom a degree of incapacitation has an adverse effect on their abilities to carry out their duties [27] or even to continue with their career. Pilots, especially, are subjected to close medical scrutiny throughout their flying career but even so there seems to be no general recognition of the prevalence of AS. Surveys of pilots and cabin crew have been carried out [24,28,29,30]. Somers has reported assessments of flight crew after exposure to fumes [31] and Heuser assessments of flight attendants [32]. These data need to be carefully examined for consistency in order to make proper use of them.

Perhaps the most extensively investigated individual case to date is that of a pilot who had to abandon his career because of symptoms of AS (despite never having suffered a “fume event”, in which the ingress of contamination into the cabin is so great as to be visible) and after a year spent trying to recover finally died. Thanks to his personal interest, and that of his family, in trying to elucidate the cause of his illness, extensive autopsies were carried out and extensive neurological damage was found [33], belying the occasionally voiced opinion that AS is not associated with organic damage. In these investigations, the focus was on TCP as the putative causative agent. Nevertheless, subsequent examination of the deceased pilot’s flying records yielded the result that his total TCP exposure was far lower than what would be expected to result in neurological damage [34,35]. It has been recognized that the aetiology of AS is far from clear [36].

The authors of ref. [33] did not consider the possibility of CO poisoning [37], although demyelination had already been associated with it [38] and delayed encephalopathy has also been noted as a consequence [39,40,41]. The data in ref. [33] should, therefore, be reexamined in the fresh light of CO poisoning as an alternative, or additional, causative agent of the neurological damage observed.

Neurological injury—encephalopathy [42]—is a common thread running through the reported symptoms of AS; the question is whether AS is rooted in a psychosomatic disorder or in actual organic neurological injury [43,44,45]. The results of autopsies, which have been carried out all too rarely despite a number of deaths apparently connected with the inhalation of aircraft cabin air fumes, seem to indicate organic injury [33]. Cognitive tests are very sensitive way of assessing neurological injury, and impairment has been correlated with changes in brain microstructure [46]. It has been found [47], albeit in a small survey, that the profile of the cognitive performance of pilots differs from that of the general population and resembles that of other groups exposed to organophosphates [48,49], notably pesticides, of which the chief examples in widespread use are sheep dip and sprays used in pomiculture and horticulture [50]. Coxon has presented brief case studies of neurological impairment following inhalation of contaminated cabin air [51]. A corollary of neurological injury is that symptoms affect many aspects of mental and physical function; the encephalopathy may be diffuse, and commonly is in survivors of chronic CO poisoning.

## 3. The Nature of Aircraft Cabin Air Contamination

The estimate of the exposure to TCP of the deceased pilot referred to above [34] was made possible, despite the absence of cockpit air composition measurements during the pilot’s flights, thanks to the study of in-flight cabin air composition for a hundred flights of various types of jet airliner [52]. While the study attracted some criticism, both from a technical viewpoint and because of the interpretation of the data [53,54], and could not be used to reach any conclusions regarding the source or cause of the measured contaminants due to the failure to report essential details of the aircraft engines and their maintenance histories, it nevertheless provided useful benchmark data for key contaminants, which are summarized in Table 1, during routine flights in which no fume events were reported. (Technical criticisms of the methodology of the measurements included the use of Tenax TA, not well suited to TCP, as the sorbent in the pumped air samplers and that whenever a reading was below the detection limit its value was reported as zero rather than (for example) half the detection limit. Nevertheless, even though measurement of the concentration of gases, vapours and particles during a “fume event” would have been of especial interest no fume event occurred during the study; given the estimated frequency of occurrence of fume events [55,56,57] this was not surprising; experienced pilots know how to generate fume events on the ground, however, and this expertise was offered to the study’s investigators but they declined to take it up [58].) Corroboration of direct measurements of the cabin air has been provided by analysis of swab samples from the interior surfaces of aircraft cabins, the isomeric composition of which matched that of the corresponding engine oil [59].

A notable feature of these data was the relatively high proportion of tri-ortho-cresylphosphate (ToCP) to total TCP [63]. This is significant because it has been established that isomers of TCP containing the ortho substituent are far more (neuro)toxic than the others [64]. For this reason, while commercial TCP is always a mixture of isomers (it would simply be too costly to purify them), its synthesis is tuned to minimize ortho content. Nevertheless, the actual ratio of ortho-TCP to the total found in the aircraft cabin air was far higher than expected from the composition of the TCP supplied as an ingredient to the turbine oil. In apparent contradiction to Henschler’s comprehensive studies using living animals, cell culture experiments showed that the different isomers were roughly equivalent with respect to neurotoxicity [65].

The inability to clearly infer that TCP was the causative agent for the observed neurological damage engendered interest in other possible chemical causative agents. Toluene is a known neurotoxin but has not received much attention as a possible causative agent for AS, despite its ubiquity (Table 1). Carbon monoxide, on the other hand, while perhaps better known for its acute respiratory toxicity, also induces a variety of cognitive deficits covering those that have been implicated in AS [20]. It is noteworthy that the measured in-flight CO concentration is 3–4 orders of magnitude greater than the concentration of TCP. Expressing the concentration in molar units would bring another order of magnitude to the difference.

## 4. Health Effects of CO and TCP

Carbon monoxide is a very well known human toxin [66,67,68]. Perhaps the least understood aspect of CO toxicity is the effect of chronic, low-level exposures barely above typical instrumental detection thresholds (i.e., of the order of 1 ppm, although some small modern portable instruments do better), which is what is mostly encountered in the aircraft cabin ([69]; Table 1).

Although such exposures are well below workplace exposure limits, the aircraft cabin atmosphere is very different from that of a workplace at sea level. Three salient differences are (i) oxygen partial pressure is only about two thirds to three quarters of the corresponding pressure at sea level because the entire aircraft cabin is pressurized typically to an altitude of 7000 feet above sea level, (ii) the atmosphere is very dry, with a relative humidity of typically 10%, and (iii) egress in the event of malaise is not possible, although an independent supply of pure air from small cylinders is available for aircrew in an emergency.

Since measurements show that several toxins are simultaneously present, the question of possible synergies arises. If present at all, *a priori* they may be adverse or beneficial and must be assessed individually. Such assessment is rarely carried out, because experimentally evaluating even all possible binary interactions between the substances listed in, for example, ref. [61] is a daunting, impracticable task. Crane studied possible synergy between inhaled acrolein and CO and found none [70]. Ramsden pointed out that since the cytochrome P450 enzymes involved in detoxifying organophosphates are haem proteins, it is rather likely that they are inhibited by CO [71]; such inhibition has recently again been observed [72,73].

A decade ago it was assumed that AS was caused by exposure to tricresyl phosphate [74]. The exclusive role of TCP was vigorously asserted by many researchers. The biochemical reasoning was superficially plausible: isomers with one or more ortho cresyl groups are metabolized in the liver into 2-(o-cresyl)-4H-1,3,2-benzodioxaphosphoran-2-one (CBDP; also known as cresyl saligenin phosphate) [75]; the enzymes involved and their genes have been identified [76]. This is consistent with Henschler’s observations (but not with Duarte et al’.s observation that all the isomers are roughly equipotently toxic to cell cultures [65]). Other isomers are expected to be indirectly toxic because they are scavenged by butyrylcholinesterase (BChE) in the blood [77,78,79], hence they diminish the availability of BChE for scavenging the ortho isomers, so more reach the liver and more CBDP is produced than otherwise. The interaction of CBDP with BChE (the other physiological roles of which are presently unknown [80]) is useful as an assay for previous exposure to tricresyl phosphate [81]. Presumably, many other enzymes form adducts with CBDP, which accounts for the complexity of its aetiology [36]. The discrepancy between the symptoms of AS and those characteristic of TCP poisoning seem not, however, to have been adequately perceived, while the much closer match between AS and known sequelae of chronic CO poisoning has hitherto received scant attention.

Another aspect is the interaction of tricresyl phosphate and its derivatives with lipids [82]. The demyelination associated with organophosphates [83] and AS [33] is consistent with the subtleties of the interactions of the isoforms of myelin basic protein (MBP) [84]. TCP has long been known as a demyelinating agent [85]. (Note a recent report by Wang et al. on the amelioration of organophosphate-induced demyelination by miconazole [86].) Demyelination is also associated with CO poisoning [38].

Due to its low toxicity (albeit to some extent possibly compensated by the higher concentration in cabin air [22]), the metabolism of TBP has not received much attention. Suzuki et al. investigated it in rats [87] and found no evidence for problematic metabolic products. Subsequent investigations of TBP administered to hens found no evidence of nerve damage [88].

Significant quantities of ultrafine particles (UFPs) were measured in all flights [52]. The particle counter used could detect and record particles in the range 0.02–1 μm in diameter, of which UFPs are defined as those smaller than 100 nm in diameter. The composition of these particles was not further investigated in ref. [52]. It can, however, be surmised that they include debris from the abradable coatings of the turbine blades [89]. (In a commercial flight, human beings would be shedding many particles from skin, clothes and exhalations, but the flights investigated in ref. [52] did not carry passengers. Jones et al. established that contamination of the turbine compressor with lubricating oil resulted in (presumably oil) nanoparticles dispersed in the bleed air [90].) Due to their high boiling points, it is inferred that TBP and TCP would be present as aerosols, hence might be counted by the detector as well as adsorbed in the pumped sample tubes, but there appeared to be no correlation between the occurrences of the semivolatile organic compounds and the UFPs, which must, therefore, be predominantly something else, such as the abradables. These are ceramic–metal (cermet) composites typically containing elements such as aluminium, chromium, nickel and zirconium. If these particles are in the nanometre range they would be invisible. The physiological effects of such particles are well studied [91]; the oxidative stress that appears to be engendered by a wide range of different nanoparticles [92] might be relevant here, although it is not obvious without further investigation whether such stress would tend to worsen or ameliorate the physiological impacts of the other contaminants in aircraft cabin air. Where fine particles are produced in the presence of combustion, they are likely to carry free radicals, some of which can be very long lived [93,94,95], implying potential for persistent biological damage.

What is neglected in all the toxicity studies on pure compounds is the possibility that in the hot environment of an aircraft jet engine (the temperature of the lubrication circuit may range up to 300 °C and hotspots may exceed 400 °C [96]) the lubricating oil may be pyrolysed or partially burnt (within the engine as a whole, temperatures can be much higher [97]). Used oil contains a very large number of unidentified substances not found in fresh oil (J.J. Ramsden, unpublished observations using GC/MS). It would be a daunting task to identify them and investigate their toxicities individually, which is presumably why early work on the inhalation toxicity of partly thermally decomposed oil [98] never seems to have been followed up, although there has been a considerable accumulation of work on the thermal degradation, including under pyrolytic conditions, of the synthetic polyol ester lubricants that form of the bulk of turbine lubricating oil [99,100,101,102,103,104] and of TBP [105].

## 5. Human Susceptibility to, and Recovery from, CO and TCP Intoxication

Airworthiness regulations mandate that “crew and passenger compartment air must be free from harmful or hazardous vapours and gases”. (e.g., US Federal Aviation Administration, Department of Transport, Section 25.831 (b).) “Harmful” or “hazardous” are not, however, defined, except for the explicit limit given for carbon monoxide (50 ppm). It is sometimes pointed out that this regulation is meaningless without also mandating the measurement of the concentrations of harmful or hazardous vapours and gases, but it is anyway meaningless without a proper understanding of the harm and hazard from the vapours and gases.

The investigators who undertook the series of measurements of potentially harmful or hazardous substances already mentioned [52] attempted to address this matter by comparing the results with established workplace exposure limits (WELs, see Table 1). The investigators pointed out that the concentrations they measured were lower than these limits, from which they concluded that “there was no evidence for target pollutants occurring in the cabin air at levels exceeding available health and safety standards and guidelines”. Regrettably, this conclusion is meaningless because the investigators ignored the fact that an aircraft cabin in flight is clearly out of the scope of the limits (ref. [61], pp. 28, 30, 62, 63, 65, 72), which are, it should be noted, time-weighted averages.

These limits must be regarded as provisional and, in a research context, one needs to enquire, for each substance of interest, how they came to be established. Taking the limits for toluene as an example (Table 1), the 15 min (384 mg/m^3^) and 8 h (191 mg/m^3^) values would appear to have been determined to a fantastic degree of precision, until one looks at the corresponding values expressed in parts per million (50 and 100, respectively), from which it is apparent that “convenient”, round figures have been chosen. The prevailing features of the environment and medical understanding at the time of setting a limit also play significant roles. When the 50 ppm limit for CO was adopted (in the 1960s), catalytic converters (invented by Eugene Houdry in the 1950s [106]) would have been useless for automobiles because the platinum catalyst would have been swiftly poisoned by the lead in the fuel. Hence, at that time the ambient CO in large cities was typically 10–50 ppm, mainly due to automotive exhaust emissions (unleaded petrol was introduced in the 1970s and the US Environmental Protection Agency (EPA) mandated catalytic converters on new cars in 1975).

Of course, there is supposed to be a wide safety margin, hence a predilection for neat, round numbers might be of no great consequence. On the other hand, 15 min at 384 mg/m^3^ results in a far lower inhaled quantity than 8 h at 191 mg/m^3^. Let it be supposed that these two exposures result in an equally adverse effect on human health (albeit that the qualitative nature of the effect might be different), then, taking them together, they only make sense if a detoxification mechanism is continuously operating. Thus, if *e* is a variable representing the (adverse) effect on health,

(1) $$\frac{\underset{\cdot }{e}}{\underset{\cdot }{t}}=kc-je$$

where *k* is the rate coefficient for assimilating a toxin at ambient concentration *c* and *j* is the rate coefficient for detoxification. Of course, some more complicated expression, for example involving fractional powers of *c* and *e* on the right-hand side, might better represent reality, but Equation (1) suffices to illustrate the matter. The solution, starting from e=0, is

(2) $$e=\frac{kc}{j}\left(1-e^{-jt}\right)$$

implying a monotonic increase to an asymptotic value of e=kc/j. Interestingly, for TBP the 15 min and 8 h limits are the same, whereas for ToCP the 15 min limit is three times the 8 h limit (p. 14 on p. 8 of ref. [61] states “Where no specific short-term exposure limit is listed, a figure three times the long-term exposure limit should be used”). From Equation (2) one can infer the *j* values (given in Table 2) by creating two simultaneous equations for the 15 min and 8 h conditions and equating them. For example, for TCP the equation is

(3) $$2+e^{-8j}-3e^{-j/4}=0$$

which has no analytical solution but can be solved graphically. Note that if the 15 min and 8 h limits are the same, the left-hand side of Equation (3) (simply $e^{-8j}-e^{-j/4}$) approaches zero asymptotically, implying that detoxification is infinitely fast, in which case it does not even matter what the actual limit is. This simple model neglects secondary paths to toxicity (such as the formation of CBDP from ToCP).

According to this model, when c=0, detoxification takes place with an exponential decay of *e* to zero with rate coefficient *j*. It is very problematical that at least some of the consequences of ToCP poisoning have been found to be irreversible [107]. Hence *j* in Equation (1) is zero, implying that there is, in such a case, no lower limit of safety. Such irreversibility is sometimes discussed as a cumulative effect [108].

Workplace exposure limits are, perforce, oriented to the standard working day of 8 h. Chronic exposure to much lower concentrations but lasting much longer are almost self-evidently deemed to be outside the scope of WELs and have been much less investigated. Given the onerous nature of carrying out an experimental study of such exposures, what we do know comes essentially from epidemiological studies [108]. Organophosphates seem to present a particular danger from this kind of exposure. The observed neurobehavioural impairment is unlikely to be via the classic acetylcholinesterase inhibition associated with acute intoxication.

WELs also implicitly assume that the human organism is able to completely reset itself during the 16 h following an 8 h workplace exposure to a toxin, the concentration of which does not exceed the 8 h limit. Terry has called attention to the neurobehavioural consequences of repeated exposures to concentrations far below the exposure limits [109].

Enormous metabolic variation among individual human beings (and between individuals of other species) is well attested [110]. Both *k* and *j* are likely to vary from person to person, and there is no reason to suppose that they are correlated with each other. In some cases these variations could exceed the margin of safety associated with each WEL. Williams noted that human endocrine patterns have not been studied, and this situation does not appear to have been improved since he highlighted it.

## 6. Public Health Impacts and Possible Remedial Action

Activities that are purely voluntary are generally more lightly regulated than those that affect everyone. Flying obviously falls into the voluntary category (at the same time the professionals in the industry are very highly regulated, most of all the pilots), and certain groups, such as people with heart problems, are already advised not to fly. Nevertheless, although in the early days of passenger aviation only a tiny minority of the population flew in aircraft, the numbers have been growing exponentially since its inception, with an annual growth rate of about 4%. At present, about 11% of the population are flyers and about 1% are frequent flyers [111]. (See also https://www.icao.int/Meetings/FutureOfAviation/Pages/default.aspx#:~:text=The%20most%20recent%20estimates%20suggest,GDP%20to%20the%20world%20economy (accessed on 16 April 2025)). If this trend continues, soon a majority of the population will rank as flyers. The COVID-19 pandemic sharply decreased passenger numbers and gave a concomitant boost to online meeting software, but passenger numbers are climbing again even though there has been a permanent increase in the popularity of online meetings. If one accepts that governments have a role to play in maintaining public health (including by means of sanitation, etc.), it follows that governments also have a duty to ensure that airline passengers are not subjected to adverse health affects. Hence the stringent safety requirements imposed on aircraft operators. Of relevance to AS is the provision (from US Federal Aviation Administration, Department of Transport, Section 25.831 (b); identical wording is found in EASA CS-25):

Crew and passenger compartment air must be free from harmful or hazardous concentrations of gases and vapours.

If an aircraft does not comply with the provision then it is formally not airworthy. There is, however, ambiguity regarding what constitutes a harmful or hazardous concentration, except in the case of carbon monoxide, for which its concentration is mandated (p. 1 following the above) to not exceed 50 ppm. The exact wording is “Carbon monoxide concentrations in excess of 1 part in 20,000 parts of air are considered hazardous. For test purposes, any acceptable carbon monoxide detection method may be used”. Surprisingly, no CO sensor seems to be routinely used in large aircraft, hence all that can be said about CO concentrations is what has been gleaned from a very small (in comparison with the total number of daily flights) number of samples. It is more technically challenging to make a compact TCP sensor for routine use and none are currently available.

“Hazard”’ denotes the possibility of harm; the actual risk depends on the product of hazard and exposure [112]. A concentration of 50 ppm of CO (58 mg/m^3^) for the entire duration of a 60 min flight leads to only one tenth of the exposure accumulated during a 10 h flight (the 15 min terrestrial workplace exposure limit is 100 ppm—Table 1). Should any explicit limit be set to exclude harm for any passenger allowed on board for the longest possible commercial flight? Furthermore, while a simple definition of “exposure” is simply the product of concentration—which equates to a certain number of molecules per unit time impinging on a surface—and time, the actual quantity entering the lungs of an individual human being is more relevant, which depends on lung capacity, breathing rate, etc.; breathing rate, in turn, depends on an individual’s metabolic requirements (a seated, sleeping passenger will have a much lower metabolic rate than a cabin crew member swiftly moving around, or a seated passenger engaged in some difficult mathematics).

It is generally considered to be an established principle of toxicology that every chemical is associated with a level of exposure below which no adverse effects will arise (for those substances essential to life, too little is of course also harmful). For the vast majority of chemicals circulating in the world economy these levels are unknown. The small (relative to the total) number of these chemicals listed in publications such as ref. [61] represents those substances for which, initially, some circumstantial evidence indicated human toxicity, whereupon further investigations were carried out—usually, on ethical grounds, not on actual human beings but on laboratory animals; studies of actual cases of intoxication also provide input. For a complex exposure, such as that arising from inhalation of aircraft cabin air, single-substance limits may be misleading since the biochemical variability among individuals increases exponentially with respect to the number of relevant substances. Based on present knowledge, it seems reasonable to assert that there are at least two—CO and TCP—and that their effects may be modulated by UFPs, low humidity, hypoxia, etc. Further complicating features include the contrasting levels of activity, both mental and physical, among aircraft occupants, which will significantly influence metabolic rates and hence the metabolism of individual substances. Neurotoxins are particularly difficult to assess, other than for acute effects such as paralysis, because some basic intracellular mechanism such as the retardation of axonal transport may have many neuronal consequences, including cognitive deficits [108,109,113]. Such consequences are likely to amplify the metabolic individuality already recognized [110]. A corollary is that it is probably impossible to set a practicable standard such that everyone is protected.

Governments have a complex relationship with the aerospace industry. Its manufacturing aspects are much sought after because they demand the highest levels of technology and hence their presence in the country strengthens the manufacturing sectors of the economy (cf. Hirschmann’s “maintenance compulsion” idea, whereby the effort required to master advanced technology entrains and advances the entire economy [114,115]). The airlines themselves are associated with prestige, as evinced by many countries aspiring to have a “national” carrier (even if it is not actually owned by the State) and doing all they can to maintain it even well beyond the bounds of economic rationality. The prestige and growing popularity of flying among the general public creates high elasticity for flight ticket demand, much as is the case with petrol for motoring, hence they can be highly loaded with various taxes and provide a lucrative and easily collectable source of revenue, which also accrues from the many jobs in the sector. In fact, the only voice countervailing the continuous growth of the industry is that of the environment, which has resulted in some restrictions on permitted hours of airport operation, runway construction and so forth.

The extent of regulation is as much an ideological question as one to be decided by rational thinking. The 1978 US deregulation act was felt to be in line with libertarian, free-market principles with little thought for the consequences. Admittedly, the more complex the technology, the harder it is to foresee the consequences of a change. As far as we can tell, bleed air is of a satisfactory quality for most people if the entire system is well maintained. The enormous cost pressures now imposed on airlines in the free market has led to this quality having been sacrificed—doubtless not intentionally, but nonetheless effectively. At the same time, there has been a gradual evolution to increase the bypass ratio of modern jet engines, meaning that it is no longer particularly economical to bleed air off them, which has in itself suggested a relatively straightforward design modification, namely to bleed air off the bypass [116]. This could do much to eliminate the contamination problem. The increase in the bypass ratio has also driven the return to the older system of separately compressing air taken indirectly from the exterior, as in the Boeing 787 (“Dreamliner”).

Nevertheless, the B787 only presently constitutes about 5% of the total number of aircraft now flying, hence in order to eliminate the health hazards from contaminated bleed air, something needs to be done that can be enacted more swiftly than the gradual replacement of aircraft. (At first sight it may seem puzzling that the lead of the B787 has not been followed by other manufacturers and is not pursued with greater vigour by Boeing. The B787, however, incorporates many innovations, including the extensive use of composite materials in the primary structure, more electric (instead of pneumatic) on-board systems—bringing a new set of problems associated with lithium-ion batteries—and sophisticated aerodynamic and engine technologies. A striking result of all these innovations is a 20–25% reduction in fuel consumption. But these are all difficult technologies to master.) Possible actions to reduce the health impacts from breathing contaminated cabin air include (cf. an earlier list given in ref. [18] and the actions suggested by Jim McAuslan in the closing speech of the BALPA conference [5])—presented in no particular order:

1. Greatly diminish the amount of flying;
2. Phase out bleed air by using separate compressors (as in the B787);
3. Redesign to bleed air off the bypass [116];
4. Mandate the installation of sensors to continuously monitor toxic gases and vapours in the aircraft cabin;
5. Install scrubbers to remove TCP, CO and possibly other toxins, e.g., based on photocatalysis [117];
6. Advise flyers, especially aircrew and frequent flyers, and those who experience jet lag or germane symptoms, to undertake a medical check specifically for susceptibility to AS or to whatever underlying cause AS can be reduced to. A standard test could be mandatory, much as was the case during the COVID-19 pandemic, but such a test still needs to be devised;
7. Reintroduce strict regulation of the industry, enabling maintenance to revert to what was usual practice prior to 1978 (this implies a multifold increase in operating costs and, concomitantly, in the price of air tickets);
8. Generally increase awareness of the problem;
9. Identify any gaps in understanding the physiology of AS and work on them, in particular in order to devise appropriate tests for susceptibility (cf. 6. above) and better therapies for recovery;
10. Mandate airlines to provide, or encourage passengers to provide for themselves, masks to eliminate the worst contaminants found to be present in inhaled air using adsorbents based on sophisticated nanomaterials. If sensors are installed on the aircraft, passengers and aircrew could be advised to don the masks if the concentration of any one contaminent, or some combination thereof, exceeds a certain threshold.

The apparent paradox of AS is the variability of outcomes. The presence of toxins, albeit at low levels (Table 1) is not disputed; what is disputed is whether these levels are harmful or hazardous to health. Clearly they are for some people. Fume events are a different matter. Toxin levels well exceed workplace exposure limits [60] and, unless a flight is swiftly landed, which is not usually the case (and which may not even be possible when crossing a great ocean), exposure may continue for several hours. Fume events are rare and their frequency as a proportion of all flights may not be changing (continual advances in oil seal technology may be roughly compensated by the continuing pressure to reduce maintenance costs, hence increasing the probability of oil seal failure, which is the most probable proximate cause of a fume event), but if the number of flights is continually increasing, they will become absolutely more common. A much greater proportion of the population must be susceptible to suffer harm from a fume event than from chronic, low-level exposure (which for most people may not be harmful [35]), but not everybody; in any discussion about AS, there will often be someone who remarks that he or she has experienced his or her “fair share of fume events” without adverse health impacts. A proper appreciation of biochemical variability does not make this at all paradoxical. Burdon et al., in an extensive review of AS and its management, have drawn attention to a finding that “six out of twelve jet airline passengers tested positive for ToCP without showing toxic symptoms” [118].

## 7. Conclusions

Three sets of phenomena need to be reconciled:

1. A large number of aircrew, and a fair number of frequently flying passengers, suffer from the collection of symptoms conveniently labeled “aerotoxic syndrome”
2. Relatively (with respect to the corresponding terrestrial workplace exposure limits) low levels of hazardous contaminants (focusing on carbon monoxide, tricresyl phosphate and ultrafine particles) are found on a significant proportion of flights;
3. Fume events are expected to result in the concentration of at least one, or possibly more, key toxins well exceeding even the 15 min terrestrial workplace exposure limit [60], and the exposure may well last much longer than 15 min;

and all must be considered in the context of the biochemical variability of individual human beings (including different respiration rates). For a pilot, a salient question may well be “what is my risk of incapacitation during a critical flight phase?”; for cabin crew it might be “what is the risk of developing debilitating illness in the course of my employment?”; and for passengers, “what is the risk of being made ill as a result of this flight?”. Although, conventionally, we all have the same risk aversion [119], in reality we do not. Furthermore, the hazard presented by a given concentration of cabin air contaminants depends on the individual’s susceptibility. More effort therefore needs to be expended on quantifying these risks if a rational approach to managing them is to be taken.

Susceptibility is likely to have a genetic determinant; for example, paraoxonase 1 (PON1) status is indicative of susceptibility to intoxication by certain organophosphates [120,121]. Suitable tests and algorithms for estimating susceptibility to intoxication by aircraft cabin air need to be devised. In the absence of good predictive tests of susceptibility, frequent flyers and aircrew are advised to be highly alert to the onset of postflight symptoms and, should they occur, to be prepared to significantly diminish their flying hours on bleed-air aircraft. (At the same time, it might be therapeutic to increase flying hours on bleed-free aircraft, since the reduced pressure at altitude should help to detoxify the body.) On the basis of what we already know, it seems sensible to assume CO poisoning in the first instance and to arrange appropriate testing with all possible urgency, undertaking appropriate treatment depending on the results of the tests.

Greater efforts should be made to improve the quality of aircraft cabin air, including lowering the present 50 ppm CO limit. This will likely result in a substantial increase in air fares. Presently, achieving “climate-neutral” aviation is expected to result in a threefold increase [122]. Taken together, we might anticipate a roughly tenfold increase in fares, which would doubtless result in a very substantial decrease in the number of people flying.

Although AS is not at present universally recognized as an occupational disease, this should not occasion great surprise if one examines the history of occupational safety and health. A milestone in the UK was the Robens report [123], in which the author wrote, “The accelerating rate of technological change, coupled with the difficulties experienced in amending or revoking old regulations, creates a situation in which the maintenance and updating of a corpus of legislation of this type, size and complexity is an endless and increasingly hopeless task”. Here is another paradox: technology advances exponentially but legislative ability to match it is slowing down. Furthermore, syndromes in general tend to be more controversial than definite diseases, a good example being autoimmune deficiency syndrome (AIDS), which even today is not universally recognized as a disease. In due course, AS may be fully recognized as an (occupational) disease, but hopefully by then its incidence will be diminishing.

Meanwhile, we shall doubtless see an evolving combination of the factors enumerated at the end of Section 6 enacted to mitigate the adverse consequences. Hopefully, the frequency of fume events will diminish, as should the low chronic concentrations of neurotoxic contaminants; simultaneously, tests for susceptibility will be improved, hence fewer people should be adversely affected. Meanwhile, an immediately implementable mitigating action for passengers is simply to have a mask with an appropriate adsorbent ready for use in the event of a fume event or other incidents of elevated CO, etc. Additionally, by raising general awareness of the matter among aircrew, passengers and their healthcare providers, symptoms of early-stage AS will be sufficiently apparent to prompt remedial action, starting with screening for CO poisoning, the at least temporary cessation of flying and prompt, appropriate medical treatment for severe cases.

## Figures and Tables

**Table 1:** Concentrations of selected toxins measured in aircraft cabin air [52].

	Ref. [52]/μg m^−3^				Ref. [60]	Ref. [61]/mg m^−3^	
Compound	${\mathrm{Mean}\;}^{a}$	s.d.	Max.	F ^*b*^	F.E. ^*c*^	15 ${\min\;}^{d}$	8 h *^e^*
CO	${\mathrm{1150}\;}^{f,g}$	–	>6000	94	–	117	23
Toluene	14	21	170	99	–	384	191
TBP	1.07	1.96	22	73	–	5	5
Total TCP	0.22 (1.0 *^h^*)	2.1	38	25	1500	–	–
ToCP	0.07	0.88	23	14	500	0.3	0.1
UFP i	$10^{10}$	–	$10^{12}$	100	–	?	?

*^a^* Arithmetic mean, with measurements below the instrument detection limit being assigned a value of 0.0. *^b^* Percentage of flights in which the compound was detected during at least part of the flight. *^c^* Estimated concentration/μg m^−3^ in a fume event. Note that “fume” signifies “solid particles generated by chemical reactions or condensed from the gaseous state, usually after volatilization from melted substances. The generation of fume is often accompanied by chemical reaction such as oxidation or thermal breakdown” (ref. [61], pp. 25, 47). *^d^* Sometimes called the safety limit. *^e^* Sometimes called the health limit. *^f^* Median. *^g^* Significantly higher values were measured by Queen on the BAe 146 aircraft [62]. *^h^* Arithmetic mean of those flights in which the compound was detected. *^i^* Unit is m^−3^. Note that median is given rather than mean in column 2.

**Table 2:** Values of the detoxification rate parameter estimated from WELs [61].

Compound	Value/h^−1^
CO	0.89
Toluene	2.75
TBP	∞
TCP	–
ToCP	1.62

## Data Availability

All data are already included in the article.
